# Supplementary material for: Venomix: a simple bioinformatic pipeline for identifying and characterizing toxin gene candidates from transcriptomic data
Source: PeerJ. 2018 Jul 31;6:e5361. doi: 10.7717/peerj.5361 (PMC6074769; doi:10.7717/peerj.5361)
Supplement: Supplemental Information 3 [file peerj-06-5361-s003.gz › FinalOutput_E-6/Kunitz-type_serine_protease_inhibitor_homolog_dendrotoxin_K_154/finaltree.pdf]

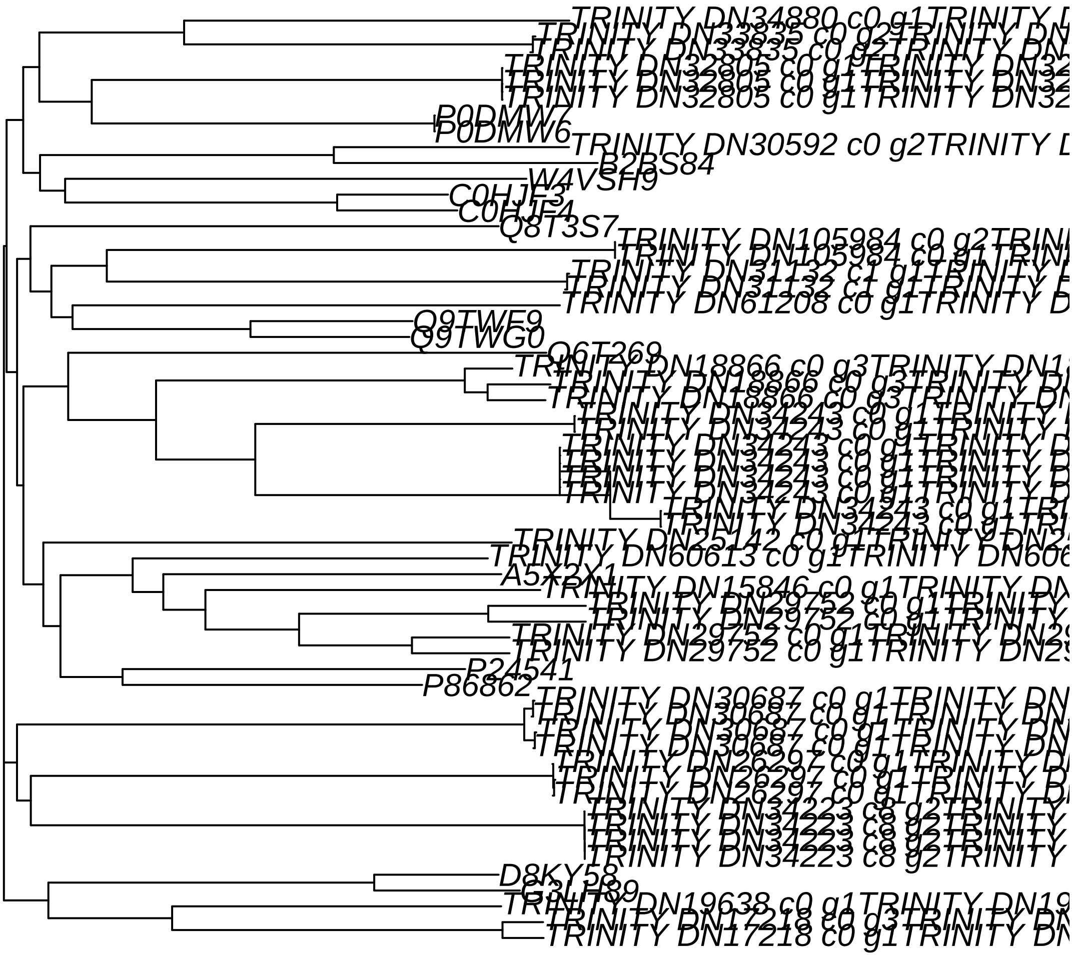

P0DMW7
P0DMW6
B2BS84
W4VSH9
C0HJF3
C0HJF4
Q8T3S7
Q9TWF9
Q9TWG0
Q6T269
A5X2X1
P24541
P86862
D8KY58
G3LH89
